# Role of neuropsin in parvalbumin immunoreactivity changes in hippocampal basket terminals of mice reared in various environments

**DOI:** 10.3389/fncel.2014.00420

**Published:** 2014-12-10

**Authors:** Harumitsu Suzuki, Dai Kanagawa, Hitomi Nakazawa, Yoshie Tawara-Hirata, Yoko Kogure, Chigusa Shimizu-Okabe, Chitoshi Takayama, Yasuyuki Ishikawa, Sadao Shiosaka

**Affiliations:** ^1^Division of Functional Neuroscience, Nara Institute of Science and TechnologyIkoma City, Nara, Japan; ^2^Department of Anatomy 2, Ryukyu University Faculty of MedicineRyukyu, Japan; ^3^Department of Systems Life Engineering, Maebashi Institute of TechnologyMaebashi, Gunma, Japan

**Keywords:** KLK8, neuregulin1, ErbB4, hippocampus, interneuron, GABA, synaptic plasticity

## Abstract

*In vitro* approaches have suggested that neuropsin (or kallikrein 8/KLK8), which controls gamma-aminobutyric acid (GABA) neurotransmission through neuregulin-1 (NRG-1) and its receptor (ErbB4), is involved in neural plasticity (Tamura et al., 2012, 2013). In the present study, we examined whether parvalbumin (PV)-positive neuronal networks, the majority of which are ErbB4-positive GABAergic interneurons, are controlled by neuropsin in tranquil and stimulated voluntarily behaving mice. Parvalbumin-immunoreactive fibers surrounding hippocampal pyramidal and granular neurons in mice reared in their home cage were decreased in neuropsin-deficient mice, suggesting that neuropsin controls PV immunoreactivity. One- or two-week exposures of wild mice to novel environments, in which they could behave freely and run voluntarily in a wheel resulted in a marked upregulation of both neuropsin mRNA and protein in the hippocampus. To elucidate the functional relevance of the increase in neuropsin during exposure to a rich environment, the intensities of PV-immunoreactive fibers were compared between neuropsin-deficient and wild-type (WT) mice under environmental stimuli. When mice were transferred into novel cages (large cages with toys), the intensity of PV-immunoreactive fibers increased in WT mice and neuropsin-deficient mice. Therefore, behavioral stimuli control a neuropsin-independent form of PV immunoreactivity. However, the neuropsin-dependent part of the change in PV-immunoreactive fibers may occur in the stimulated hippocampus because increased levels of neuropsin continued during these enriched conditions.

## Introduction

The secretory serine protease neuropsin is considered to be involved in activity-dependent neural plasticity such as the early phase (E-) of long-term potentiation (LTP) and kindling epileptogenesis (Chen et al., 1995; Okabe et al., 1996; Tamura et al., 2006). The identification of neuropsin’s substrates (neuregulin-1 (NRG-1), fibronectin, vitronectin, and L1cam) have aided in elucidating the understanding of the function of neuropsin (Shimizu et al., 1998; Matsumoto-Miyai et al., 2003; Tamura et al., 2012). Hippocampus of the neuropsin-deficient mice exhibited reduction in a mature mushroom-type synapses and increase in a small immature synaptic terminals labeled by L1cam antibody and the changes were reversed by the intraventricular application of recombinant neuropsin (Nakamura et al., 2006). In addition, deficient mice exhibit a disappearance of E-LTP (Komai et al., 2000; Tamura et al., 2006), and the synaptic association between two independent inputs of the Schaffer-collateral pathway, which is the basis of hypothetical synaptic tagging and which was reversed by a bath application of recombinant neuropsin (Ishikawa et al., 2008, 2011). These results suggest that neuropsin is involved in synaptic modulation during the early stages of neural plasticity by cleaving (or modifying) the extracellular domain of target proteins.

Our recent study has suggested that neuropsin induces the neural activity-dependent release of the NRG-1 ligand, regulates interaction of an NRG-1 with ErbB4 into parvalbumin (PV)-positive neurons, and as a result, strengthens gamma-aminobutyric acid (GABA) neurotransmission (Tamura et al., 2012). It is well known that ErbB4 in the PV-positive GABAergic interneurons triggered phosphorylation by cleaving NRG-1 in an LTP-dependent manner (Longart et al., 2007; Vullhorst et al., 2009; Buonanno, 2010; Shamir et al., 2012; Tamura et al., 2012). Thus, neuropsin may play a role only in the early period (less than a few hours) of inhibitory signaling and regulate hippocampal pyramidal neurons through GABAergic neurotransmission. The GABAergic system in the hippocampus is involved in synchronization and gamma oscillation (Klausberger et al., 2005; Sohal et al., 2009; Volman et al., 2011; Hou et al., 2014) and possibly in cognitive functions in humans and animals. Several studies have shown a functional relevance of these molecules in human cognitive function; NRG-1, ErbB4, and neuropsin are all risk factors for schizophrenia/bipolar disorder (Britsch et al., 1998; Stefansson et al., 2003; Corfas et al., 2004; Izumi et al., 2008; Mei and Xiong, 2008). In addition, animal studies have supported this because both ErbB4- and neuropsin-deficient mice exhibit impaired working memory and memory acquisition (Tamura et al., 2006; Wen et al., 2010).

Multiple lines of evidence have suggested that neuropsin is involved in synaptic plasticity. However, the role played by neuropsin in the hippocampus of voluntarily behaving animals remains unknown. In the present study, we examined: (1) whether an enriched environment (EE) stimulated the induction of neuropsin and PV immunoreactivity in the hippocampus; and (2) whether neuropsin regulated PV immunoreactivity in mice reared in familiar and EEs.

## Results

**Figure 1:**
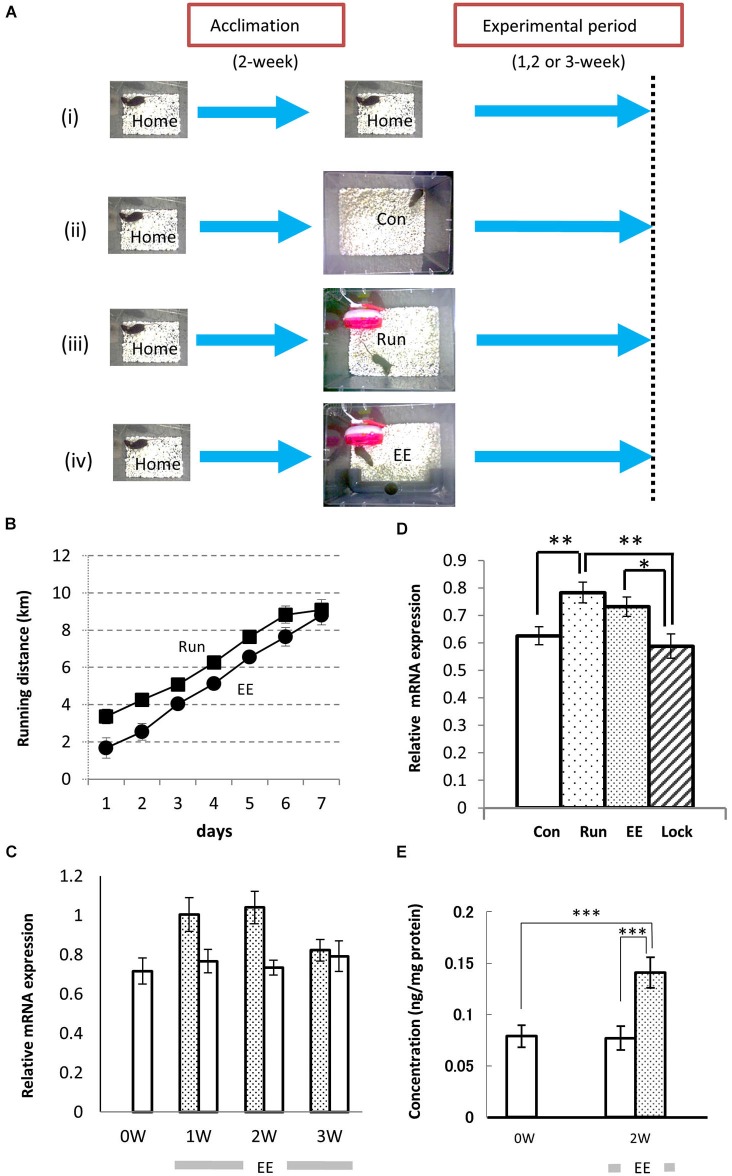
**Environmental stimuli upregulate the levels of expression of neuropsin mRNA and protein in the hippocampus. (A)** The enrichment protocol was applied to voluntarily behaving mice. After a 2-week acclimation in the home cage, the mice were maintained in the same home cage (i) or transferred to a large cage (28 cm × 33 cm × 16 cm; Con, (ii), a large cage containing only a plastic running wheel (Run; iii), a large cage with a running wheel and a plastic opaque tube (EE; iv), or a large cage with a nonrevolving wheel, which was fixed with glue, and a plastic tunnel (Lock). **(B)** The running distances (counts of rotation/day × circumference of wheel) were increased daily both in the Run (Filled squares) and EE (Filled circles) rich environments. **(C)** The time course of the change in neuropsin mRNA during longer experimental periods (1–3 weeks). One week (1 W) or 2 weeks (2 W) of exposure to the environmental enrichment (EE) resulted in an increase in neuropsin mRNA. After 3 weeks (3 W), the increase in the levels of expression of neuropsin mRNA in the hippocampus of EE mice returned to the Con level. Statistical significance was determined by a one-way analysis of variance (ANOVA) between the control (Con: open bars) and EE (hatched bars) groups (*n* = 7). *p < 0.05. **(D)** Significant increases in the levels of neuropsin mRNA expression in the hippocampus were seen in both the EE and Run groups during the 1-week experimental period. Note that no increase in mRNA was found when the running wheel was locked (Lock; *n* = 7). The error bars indicate standard error of the mean (SEM). The levels of statistical significances were *p < 0.05 or **p < 0.01. **(E)** The enzyme-linked immunosorbent assay represents a change in neuropsin protein levels during the experimental period. A significant increase in the immunoreactivity of neuropsin was found in the EE group. ***p < 0.005. The experimental period was 2 weeks (2 W).

To analyze the effects of neuropsin on hippocampal neurons in voluntarily behaving mice, we first examined neuropsin mRNA and protein levels in animals reared in familiar and EEs. Prior to the experiment, a single mouse was reared in a familiar cage (10- × 20- × 14-cm^3^ home cage; **Home**) for acclimation (Figures 1Ai–iv). Furthermore, two control and two types of enriched conditions were set up (Figures 1Ai–iv), and the mRNA levels of the hippocampi of the animals reared in each of the environments were analyzed by real-time polymerase chain reaction (RT-PCR). The rearing conditions were as follows (Figure 1A): (i) mice were kept in the home cage during all of the experimental periods; (ii) mice were transferred into a large cage (28 × 33 × 16 cm^3^; **Con**); (iii) mice were exposed to the enriched condition of a running wheel in the large cage (**Run)**; and (iv) mice were exposed to another enriched condition of a running wheel and a plastic tunnel for hiding in the large cage (**EE**). With both behavioral stimuli, the running distance was calculated by counts of rotation/day × circumference of the wheel (Section Materials and Methods). Mice exposed to both enriched conditions ran longer daily and reached approximately 9 km/day after 1 week (Figure 1B).

### Neuropsin mRNA and protein levels were increased in the hippocampus by environmental stimuli in the voluntarily behaving mice

After mice were transferred into an EE (Figure 1Aiv) or large control cage (Con; Figure 1Aii), the time course of the expression of neuropsin mRNA was determined by quantitative PCR during 1-, 2- or 3-week rearing. A significant increase was observed after 1 and 2 weeks, and it returned to basal levels after 3 weeks (Figure 1C). Furthermore, we compared the levels of expression of neuropsin mRNA in the hippocampi of mice reared in the two enriched conditions, Run and EE, after 1 week. In both cases, the levels of neuropsin mRNA were significantly upregulated (Figure 1D). However, when the wheel was locked with a stopper till it stopped rotating (Lock), the levels of expression were the same as the control level (Con) (Figure 1D). The changes in neuropsin were further quantified by an enzyme-linked immunosorbent assay (ELISA) for neuropsin protein. Two weeks of rearing of the mice in EE resulted in a significant increase in neuropsin immunoreactivity (Figure 1E). These results suggested that environmental stimuli contribute to an upregulation of neuropsin expression.

### No significant changes were found in the total cell number of PV-immunoreactive interneurons in the neuropsin-knockout (NPKO) mice reared in the familiar cage

**Figure 2:**
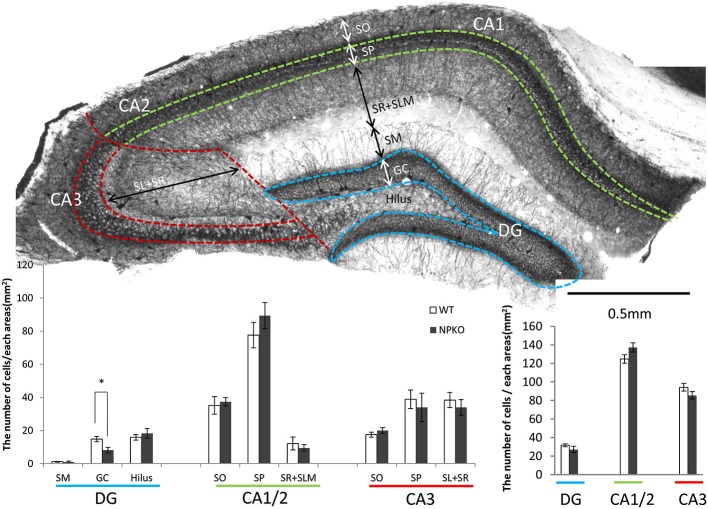
**The quantitative analysis of parvalbumin (PV)-immunoreactive neurons between wild-type (WT) and neuropsin-deficient (NPKO) mice. (A)** Parvalbumin-immunoreactive neurons and fibers in a coronal section of mouse hippocampus. A 3,3′-diaminobenzidine reaction revealed thick positive nerve terminals surrounding the CA1-3 pyramidal neurons and granular neurons. The blue, green, and red broken lines on a whole section represent the boundaries dividing the sublayers and subfields of the hippocampus. Scale bar: 0.5 mm. **(B)** Number of PV-immunoreactive neurons in each sublayer in the dentate gyrus (DG; blue line), CA1 and two subfields (CA1/2; green line), and CA3 subfield (CA3; red line) of the hippocampus (*n* = 4; the average from 10 slices from each mouse were calculated). Open bar: WT, Closed bar; NPKO mice. **(C)** Total number of PV-immunoreactive neurons in each area. There was no difference in the total positive cell number between WT (Open bar) and NPKO (Closed bar) mice. Abbreviations: CA1, CA1 subfield; CA2, CA2 subfield; CA3, CA3 subfield; DG, dentate gyrus; GC, granule cell layer; SL, stratum lucidum; SM, stratum moleculare; SO, stratum oriens; SP, stratum pyramidale; SR, stratum radiatum. The asterisks indicate significance: *p < 0.05. The error bars indicate SEM.

Because neuropsin interacts with PV-immunoreactive neurons through ErbB4 signaling, as shown by Tamura et al. (2012), the hippocampal PV-immunoreactive neurons were examined in the neuropsin-deficient mice. In our earlier study, no remarkable changes in PV-immunoreactive cell number were found in the pyramidal cell layer of the CA1 subfield (Hirata et al., 2001). To confirm the results and extend the findings to other hippocampal subfields, we performed thorough quantitative analyses in each layer of the dentate gyrus (sectioned by broken blue lines of Figure 2A), CA1/2 (sectioned by broken green lines of Figure 2A), and CA3 (sectioned by broken red lines of Figure 2A). In agreement with our previous study, negligible changes in PV-immunoreactive cell numbers were observed in the granular cell layer of the dentate gyrus (Figure 2B), and the total numbers of PV cell bodies in each subfield were not changed, even in the NPKO mice (Figure 2C). In addition, no morphological changes in PV-immunoreactive cells, such as cell size or dendritic arborization, were observed in both genotypes (data not shown; Hirata et al., 2001). No significant changes in the number of GAD67-immunoreactive cell bodies were observed in the previous and present study (data not shown; Hirata et al., 2001). Therefore, PV-positive inhibitory interneurons were considered to be maintained normally even in the neuropsin-deficient mice.

### Marked decreases in the intensity of PV-immunoreactive fibers in the dentate gyrus and the CA1 and CA3 subfields were observed in NPKO mice reared in a familiar cage

**Figure 3:**
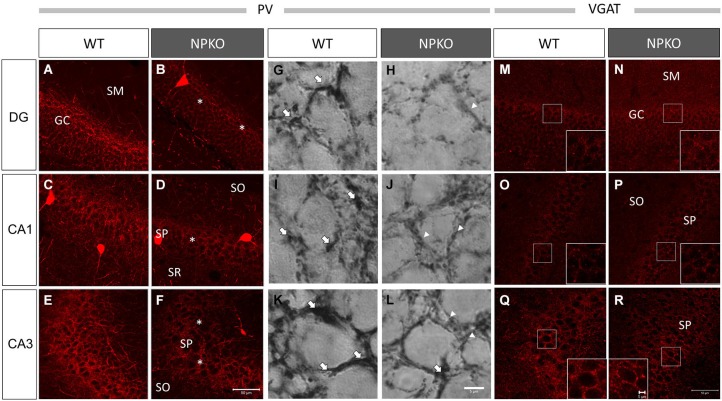
**Marked downregulation of PV immunoreactivity in the NPKO mice compared to the WT mice**. Confocal microscopic images **(A–F)** and Nomarski differential interference images **(G–L)** are presented. Dense PV-immunoreactive fiber networks were localized in the GC **(A)** and SP **(C,E)** of CA1 and CA3 subfields. The decrease in PV immunoreactivity was significant in the GC **(B)** and SP of the CA3 subfield **(F)**, and the decrease was less significant in the SP of the CA1 sublayer **(D)**. The Nomarski images show that the PV-immunoreactive fibers surrounding the granular and pyramidal cells were thick fiber bundles (Open arrows in **G,I,K**), whereas, in the NPKO mice, only weakly immunoreactive fibers remain (Open arrowheads in **H,J,L**). The scale bars indicate 50 µm **(A–F)** and 6 µm **(G–L)**. Vesicular GABA transporter (VGAT) immunoreactivity was not changed between the WT and NPKO mice. Both of the immunofluorescent images of the WT **(M,O,Q)** and NPKO **(N,P,R)** mice show similar staining patterns in DG, CA1, and CA3. Because VGAT concentrates in GABAergic nerve endings, the inhibitory synaptic apparatuses may be intact in the NPKO mice. The insets show higher magnification of the boxed areas of the photographs. The scale bars indicate 50 µm and 5 µm in the insets.The abbreviations listed in Figures 3–6 are the same as those in Figure 2.

**Figure 4:**
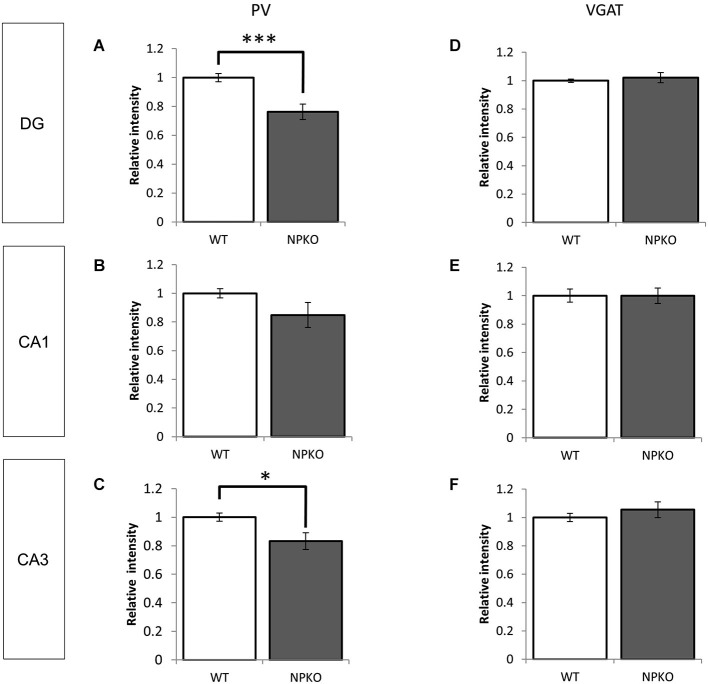
**Densitometry analyses of PV and VGAT immunoreactivities in the hippocampus of WT and NPKO mice**. The relative fluorescent intensities of PV- **(A–C)** and VGAT-immunoreactive fibers **(D–F)** were measured and compared between WT and NPKO mice. A significant downregulation of the PV-immunoreactive fibers was observed in the DG **(A)** and CA3 **(C)** of NPKO mice. *p < 0.05, ***p < 0.005. A moderate but nonsignificant reduction in the PV-immunoreactive intensity was also observed in the CA1/2 subfields (**B**; *n* = 3). Six to 11 images (200 × 200 pixels) of similar areas in the WT and NPKO mice were combined. No change in the relative intensity of VGAT immunoreactivity was observed in all observed regions (*n* = 5). Five to 14 images of similar areas in the WT and NPKO mice were combined. The error bars indicate SEM. The relative intensities of the PV and VGAT immunoreactivity were analyzed by ImageJ software.

In the hippocampal slices, paired-pulse inhibition experiments result in a reduction in GABAergic release from ErbB4-containing PV-positive neurons in the CA1 subfield of deficient mice (Tamura et al., 2012). Such physiological deficits in the GABAergic nerve terminals of neuropsin-deficient mice suggest that neuropsin controls granular and pyramidal neurons through basket and/or axo-axonic fiber networks. Therefore, we examined PV-immunoreactive nerve terminals in the granular layer of the dentate gyrus and the stratum pyramidale of the CA1-3 subfields (Figures 3A,C,E) and compared them to those in neuropsin-deficient mice (Figures 3B,D,F). The intensities of the PV-immunoreactive fibers were significantly decreased, particularly in the granular cell layer of the dentate gyrus (Figure 4A) and the stratum pyramidale of the CA3 subfield (Figure 4C) and less significantly decreased in the stratum pyramidale of the CA1 subfield (Figure 4B). The intensities of PV terminals, which were measured in randomly selected boxed areas, were significantly reduced in the dentate gyrus and CA3 subfield (Figures 4A,C). Higher-magnification photographs that were obtained under a Nomarski interference condenser showed that thick PV-immunoreactive axons surrounding the cell somas in the granular and pyramidal layers (Figures 3G,I,K, thick open arrows) were present in the wild-type (WT) mice, and these axons were mostly reduced in the deficient hippocampus (Figures 3H,J,L). However, weak punctate immunoreactivity remained in the surrounding granular and pyramidal neurons, even in the deficient hippocampus (arrowheads; Figures 3H,J,L).

**Figure 5:**
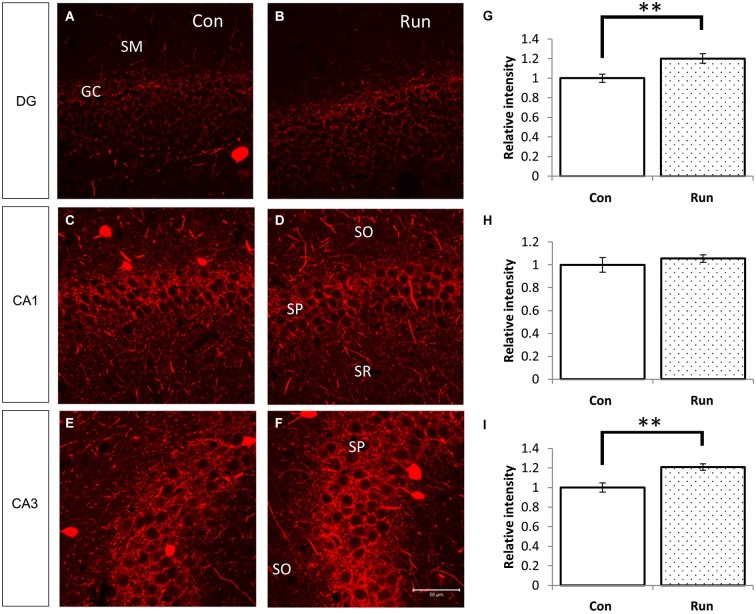
**Voluntary running upregulates PV-immunoreactive fibers in the hippocampus**. Parvalbumin-immunoreactive fibers were increased in mice that were reared in the cage with a running wheel (Run) compared to the control (Con) **(A–F)**. The hippocampi of voluntarily running mice showed higher fluorescent intensities of PV immunoreactivity in the granular layer of the DG and the pyramidal layer of CA3 **(A,B,E,F)**, and the difference in CA1 was not as remarkable **(C,D)**. Densitometry analyses of the relative intensities of the photographs of PV immunoreactivity **(G–I)**. Significant increases in the PV immunoreactivity in the DG **(G)** and CA3 **(I)** of the Run group were observed, but no statistical differences were observed between the Run and Con groups in the pyramidal layer of CA1 **(H)**. Student’s *t*-test: **, *p* < 0.01. The error bars show SEM [*n* = 4 (Con) and 3 (Run)]. Nine 200 × 200 pixel images of similar areas from each mouse were combined.

**Figure 6:**
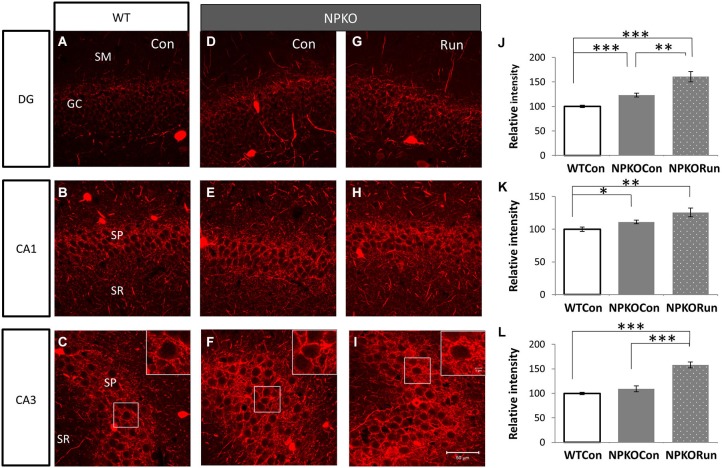
**The neuropsin-dependent form for the induction of PV immunoreactivity during voluntary running cannot be confirmed in the NPKO mice**. The induction of PV immunoreactivity in the mice reared in the cage with a running wheel (Run) was compared between the knockout (NPKO; **D–F**) and WT-type hippocampus (**A–C**; same photographs with those of Figures 5A,C,E). After transfer from the home cage to a large experimental cage (Con: Figure 1Aii), a slight increase in PV immunoreactivity was observed **(D–F)**. In the Run group, a further increase in PV immunoreactivity, even in the knockout mice, was observed in DG, CA1, and CA3 **(G–I)**. Densitometric analysis of PV immunoreactivities in the hippocampus of WT and NPKO mice **(J–L)**. Parvalbumin immunoreactivity was not impaired but rather was increased in the NPKO mice when they were transferred from the home cage into a large experimental cage (without toys). The hippocampi of mice that could voluntarily run showed even higher fluorescent intensities of PV immunoreactivity in the granular layer of the DG and the pyramidal layer of CA1 and 3 **(J–L)**. *p < 0.05, **p < 0.01, ***p < 0.005.

Furthermore, we examined whether the decreases in the densities of PV-immunoreactive fibers were caused by any axonal loss of inhibitory fibers because of the neuropsin deficiency. We examined the vesicular GABA transporter (VGAT; a transporter that mediates the accumulation of GABA into inhibitory synaptic vesicles) with immunostaining in WT and neuropsin-deficient mice. No apparent changes were observed in the three areas that were examined in the subfields of the hippocampus (Figures 3M–R, 4D–F). The results of the PV and VGAT immunoreactivities suggested that GABAergic terminal boutons maintained the inhibitory vesicles, whereas the PV immunoreactivity was reduced in the boutons in the deficient hippocampus.

### A marked increase in the intensity of PV-immunoreactive fibers was observed in mice running voluntarily

We examined PV immunoreactivity in the voluntarily running mice because neuropsin mRNA and protein levels were upregulated by transferring mice into rich environments. Parvalbumin immunoreactivity was upregulated in the granular layer of the dentate gyrus and stratum pyramidale in the CA3 subfield of the mice allowed to voluntarily run (Run) (Figures 1Aiii, 5A–I).

### Transfer to a rich environment increased PV immunoreactivity even in the NPKO mice

Because voluntary running itself induced an increase in PV immunoreactivity in the hippocampus (Figure 5), we further analyzed whether the increase was because of the effects of neuropsin on PV neurons. In NPKO mice, PV immunoreactivity was slightly increased only after transferring the mice into a novel large cage (Figure 1Aii; Con; WT vs. NPKO; Figures 6A–F,J–L). Two weeks of rearing in the large cage with the running wheel (Figure 1Aiii; Run) resulted in a marked increase in PV immunoreactivity, even in the knockout mice (WT vs. NPKO; Figures 6A–C,G–L). These results showed that at least some of the plastic changes in PV immunoreactivity by the environmental stimuli were because of a neuropsin-independent mechanism.

## Discussion

Increasing levels of PV in the hippocampal basket cells indicated a state of plasticity. Donato et al. (2013) reported that low PV content parallels enhanced memory and structural synaptic plasticity (plastic state; state for high capacity of acquisition), while high PV content parallels a low plasticity (consolidated state; state for fixation of memories). When PV cells are activated and promoted into a high PV state, these may impede hippocampal plasticity (Donato et al., 2013; Hensch, 2014). Optogenetic technology has revealed that the specific driving of PV interneurons induces enhanced gamma oscillation, which contributes to cognitive functions, such as memory formation and sensory processing (Sohal et al., 2009). In contrast, reduced PV parallels with the decreases in gamma oscillation in computational models (Volman et al., 2011), which was similar to what is observed in diseases involving cortical dysfunction, such as schizophrenia (Lewis et al., 2012). In addition, researchers have shown that physical exercise induces plastic changes in inhibitory neurons in the hippocampus. Arida et al. have reported that both 10 or 45 days of voluntary running (acute or chronic exercise) increases PV expression in cell bodies (Arida et al., 2007; Gomes da Silva et al., 2010). Thus, PV levels may correlate with the plastic state in the inhibitory system, even in the voluntarily acting mice and in subsequent cognitive behavior of the animals. In the present study, we focused on the involvement of neuropsin, a plasticity-related serine protease on PV-immunoreactive nerve terminals in the pyramidal and granular layers, when a mouse exercised (in a rich environment) and transient upregulation of the neuropsin was observed during 1- or 2-week exposure of wild mice to voluntary exercise.

Previous studies on animals in restricted conditions have demonstrated that neuropsin is involved in the physiological regulation of hippocampal and amygdaloid plasticity in exploring behavior (Tamura et al., 2006), the induction of hippocampal E-LTP* in vivo* (Tamura et al., 2006), and anxiety (Horii et al., 2008; Attwood et al., 2011). Moreover, *in vitro* studies have demonstrated that the processing of NRG-1 by neuropsin allows an interaction of NRG-1’s functional domain with ErbB4, which consequently regulates GABA_A_ receptor-dependent transmission and increases the potentiation of E-LTP (Tamura et al., 2012). The physiological impairments that resulted from the neuropsin deficiency are reversible by the addition of neuropsin protein, neuropsin-treated recombinant NRG-1 (cleaved NRG-1), or the ErbB4-activating domain of the NRG-1 peptide *in vivo* (Tamura et al., 2006, 2012; Ishikawa et al., 2008). Thus, neuropsin may be a key cleavage enzyme for NRG-1 for inducing NRG-1-ErbB4 signaling. This system controls hippocampal plasticity through a GABAergic inhibitory network in a neural activity-dependent manner. In the present study, we observed in voluntarily behaving mice that the intensity of PV immunoreactivity in the tranquil mice was significantly decreased in the neuropsin-deficient mouse, suggesting that neuropsin regulates PV immunoreactivity in mice under the relaxed condition. Therefore, the major function of neuropsin on the inhibitory neurons may strengthen the transient GABAergic transmission via ErbB4 NRG-1 receptor after continual occurrence of plasticity in the nonstressful condition.

Although we found an upregulation of neuropsin mRNA and protein in the hippocampus with an increase in hippocampal PV-immunoreactive terminals after environmental change, we did not find any detrimental effects on PV immunoreactivity in the neuropsin-deficient mouse hippocampus. Instead, we found an increase in PV immunoreactivity when mice were placed in novel environments. When mice were transferred to the unfamiliar cage, the apparent inverse effects in the neuropsin-deficient mice may be attributable to alternative signaling activating PV cells other than neuropsin. A similar phenomenon was observed in L-LTP induced by strong tetanic stimulus into the Shaffer-collateral pathway (Ishikawa et al., 2008, 2011). This case represents the existence of a neuropsin-independent form of long-term plasticity. However, we do not have direct evidence that neuropsin acts on PV immunoreactivity in the dynamic plasticity state because neuropsin deficiency did not impair PV immunoreactivity during environmental changes. However, a continual increase in neuropsin mRNA and protein in the enriched cage may demonstrate that neuropsin-dependent plasticity is hidden by the stronger and long-lasting neuropsin-independent control for PV.

In summary, in the nonstressful condition, neuropsin was involved in the control of PV networks in the hippocampus, whereas, in EEs, even with changing to a different cage, mice received strong psychological inputs. Consequently, the PV network was regulated by a neuropsin-independent system and presumably by a neuropsin-dependent system.

## Materials and methods

### Animals

A total of 130 C57BL/6J mice and 50 neuropsin-deficient mice (6–10 weeks of age) were used in this study. The neuropsin-deficient mice were generated as previously described (Hirata et al., 2001). The mice were backcrossed with the C57BL/6J strain 12 times. The mice were maintained under a 12-h light/dark cycle and given food and water *ad libitum* according to the guidelines of the Nara Institute of Science and Technology. The study was approved by the institutional animal care and user’s committee.

### Environmental stimulation

Mice were individually housed until the behavioral experiments. After 2 weeks of acclimation, the mice were transferred into each environmental setting (Figures 1Aiii,iv). The objects in the cages were maintained in the same position. For the environmental stimulation conditions, a plastic running wheel (Sanko Shokai Co., Ltd., Osaka, Japan) for free, voluntary running (Run) or a running wheel and a plastic opaque tube for a more complex, EE were placed in a large cage (28 cm × 33 cm × 16 cm). For the controls, no objects (Con) or a nonrevolving wheel, which was fixed with glue and a plastic tunnel (Lock), were placed in the large-sized cage. Moreover, some mice were kept in home cages (Home). Running distance was calculated by counting the rotation numbers × the circumference of the plastic wheels (11 cm diameter). Rotation number was checked at 10 A.M. daily, and mice that ran less than 10,000 rotations per day (less than 3.5 km) were excluded from the groups; both 8/19 and 8/18 mice were excluded from Run and EE groups, respectively.

### Tissue preparation and immunohistochemistry

Mice were deeply anesthetized with an intraperitoneal injection of 10% urethane (15 mg/kg) and perfused with 50 mL of 0.1 M phosphate-buffered saline (PBS; pH 7.4), which was followed by 100 mL of PBS containing 2% paraformaldehyde through the ascending aorta. The brains were removed and postfixed overnight in the same fixative at 4°C. The brains were then consecutively rinsed for 1 h in cold (4°C) 70%, 80%, 90%, and 95% ethanol and then placed in 100% ethanol overnight. Furthermore, the brains were rinsed through 50% and 75% mixtures of ethanol and polyester wax (VWR International LLC, Radnor, PA, USA) at 42°C and placed in 100% polyester wax two times for 1 h each. The brains were embedded in 100% polyester wax. Sections (6-µm-thin and 50-µm-thick) were cut with a microtome (Microm HM400, Microm International GmbH, Walldorf, Germany) for immunofluorescent observations and differential interference observations, respectively.

The sections were dewaxed three times by immersion in 100% ethanol and were then immersed in PBS. All immunostaining was performed with the floating method. The sections were blocked for 2 h in 5% bovine serum albumin in 0.3% Triton X-100, incubated overnight at 4°C with a monoclonal or polyclonal antiparvalbumin antibody (monoclonal, 1:3.000; Sigma-Aldrich Japan K.K., Tokyo, Japan; polyclonal, 1:1500, Abcam plc, Cambridge, UK) that was diluted with 1% bovine serum albumin in 0.3% Triton X-100 in PBS. After washing with PBS, the sections were incubated overnight at 4°C with Alexa-labeled secondary antibodies (1:500). For light microscopic and differential interference observations of the immunoreactivity, a Vectastain ABC kit (Vector Laboratories, Inc., Burlingame, CA, USA) was used and visualized by a diaminobenzidine reaction.

A polyclonal antibody for VGAT was produced by immunization as described elsewhere (Takayama and Inoue, 2010). The sections were incubated overnight with the VGAT antibody (1:400), and the same protocol as that described above was used.

### RNA extraction and real-time RT-PCR

The mice hippocampi were dissected, and total RNA was extracted with TRIzol reagent (Life Technologies, Grand Island, NY, USA). The relative levels of mRNA expression were measured by LightCycler480 (Roche Diagnostics Japan, Tokyo, Japan). Cp values were determined by the second derivative maximum method. The *glyceraldehyde-3-phosphate dehydrogenase* (*GAPDH*) gene was used as an internal control. The primer sequences for the *neuropsin* gene were (forward) 5′-CCCACTGCAAAAAACAGAAG-3′ and (reverse) 5′-TGTCAGCTCCATTGCTGCT-3′. The primer sequences for *GAPDH* were (forward) 5′-CGGGAAGCCCATCACCATC-3′ and (reverse) 5′-GAGGGGCCATCCACAGTCTT-3′. The primer concentrations were 0.2 µM for both genes. Samples were preheated at 95°C for 10 min. The PCR conditions were 95°C for 10 s, annealing temperature of 60°C for 10 s, and extension temperature of 72°C for 14 s. The sample concentration was calculated by the average of the triple measurements.

### Enzyme-linked immunosorbent assay (ELISA) for neuropsin

The dissected hippocampi were homogenized with lysis buffer [1 mM ethylenediaminetetraacetic acid, 0.5% (w/v) Triton X-100 in PBS], and the supernatants that were collected after centrifugation (15,000 rpm, Tomy Seiko Co., Ltd., Tokyo, Japan) were used as samples. An antineuropsin monoclonal antibody (clone F12; Medical & Biological Laboratories Co., Ltd., Nagoya, Japan) was adsorbed in 96-well ELISA plate (Sumiron Co., Ltd., Tokyo, Japan). After blocking with 5% skim milk in PBS, sample aliquots were incubated at 4°C overnight. An antineuropsin rabbit polyclonal antibody, peroxidase-labeled antirabbit immunoglobulin, and substrate for peroxidase 3,3′,5,5′-tetramethylbenzidine were used for detection and were measured with a microplate reader at 450 nm absorbance (Model 3350; Bio-Rad Laboratories, Inc., Tokyo, Japan).

### Observation, quantitative analysis, and fluorescent densitometry analysis of the immunostaining

Observations were performed with a LSM 710 Zeiss Axio Observer ZI confocal laser-scanning microscope (Carl Zeiss AG, Jena, Germany) that was equipped with a differential interference prism and fluorescent light source.

The number of PV-immunoreactive neurons was determined in 40 immunostained coronal sections (50-µm thickness) from four WT and four neuropsin-deficient mice. Sections that were reacted with diaminobenzidine as a chromogen were observed and photographed under a Zeiss Axioplan 2 microscope. The numbers of positive cells were counted in each area of the photographs that was encircled by the boundaries shown in Figure 2A.

For densitometry analyses, hippocampal sections from all groups were immunostained with the same reaction media of primary antibodies and Alexa-labeled secondary antibodies together. Photographs were taken under the same strength of Laser power and exposure time of Zeiss confocal microscope, and intensities of immunopositive nerve terminals were measured and compared from the photographs of WT and NPKO mice (NPKO; 3–5 mice) with ImageJ software. The 200 × 200-pixel boxes which do not contain positive cell bodies were randomly selected on photographs. Background (intensities measured at the nuclei of the pyramidal cells) was subtracted from the 200 × 200-pixel images. Five to 14 coronal hippocampal sections from each mouse were used for the analysis.

### Statistical analysis

Statistically significant differences were determined by Student’s *t*-tests, or one-way analysis of variance (ANOVA) with a Dunnet’s *post hoc* test. All of the data are presented as means ± standard errors of the mean. The number of mice is indicated by *n*, and *p* values less than 0.05 were considered statistically significant.

## Conflict of interest statement

The authors declare that the research was conducted in the absence of any commercial or financial relationships that could be construed as a potential conflict of interest.
